# Epigenetic regulatory mechanism of ADAMTS12 expression in osteoarthritis

**DOI:** 10.1186/s10020-023-00661-2

**Published:** 2023-07-03

**Authors:** Shu Yang, Xuanping Zhou, Zhen Jia, Mali Zhang, Minghao Yuan, Yizhao Zhou, Jing Wang, Duo Xia

**Affiliations:** 1 Department of Orthopedics, Hunan Provincial People’s Hospital (The First-affiliated Hospital of Hunan Normal University), No. 61, Jiefang West Road, Furong District, Changsha, Hunan 410005 People’s Republic of China; 2 Department of Orthopedics, The First-affiliated Hospital of Hunan Normal University (Hunan Provincial People’s Hospital), Changsha, Hunan 410005 People’s Republic of China

**Keywords:** Osteoarthritis, Inflammation, ADAMTS12, STAT1, METTL3, IGF2BP2

## Abstract

**Background:**

Osteoarthritis (OA) is a degenerative joint disease with lacking effective prevention targets. A disintegrin and metalloproteinase with thrombospondin motifs 12 (ADAMTS12) is a member of the ADAMTS family and is upregulated in OA pathologic tissues with no fully understood molecular mechanisms.

**Methods:**

The anterior cruciate ligament transection (ACL-T) method was used to establish rat OA models, and interleukin-1 beta (IL-1β) was administered to induce rat chondrocyte inflammation. Cartilage damage was analyzed via hematoxylin-eosin, Periodic Acid-Schiff, safranin O-fast green, Osteoarthritis Research Society International score, and micro-computed tomography assays. Chondrocyte apoptosis was detected by flow cytometry and TdT dUTP nick-end labeling. Signal transducer and activator of transcription 1 (STAT1), ADAMTS12, and methyltransferase-like 3 (METTL3) levels were detected by immunohistochemistry, quantitative polymerase chain reaction (qPCR), western blot, or immunofluorescence assay. The binding ability was confirmed by chromatin immunoprecipitation-qPCR, electromobility shift assay, dual-luciferase reporter, or RNA immunoprecipitation (RIP) assay. The methylation level of STAT1 was analyzed by MeRIP-qPCR assay. STAT1 stability was investigated by actinomycin D assay.

**Results:**

The STAT1 and ADAMTS12 expressions were significantly increased in the human and rat samples of cartilage injury, as well as in IL-1β-treated rat chondrocytes. STAT1 is bound to the promoter region of ADAMTS12 to activate its transcription. METTL3/ Insulin-like growth factor 2 mRNA-binding protein 2 (IGF2BP2) mediated N6-methyladenosine modification of STAT1 promoted STAT1 mRNA stability, resulting in increased expression. ADAMTS12 expression was reduced and the IL-1β-induced inflammatory chondrocyte injury was attenuated by silencing METTL3. Additionally, knocking down METTL3 in ACL-T-produced OA rats reduced ADAMTS12 expression in their cartilage tissues, thereby alleviating cartilage damage.

**Conclusion:**

METTL3/IGF2BP2 axis increases STAT1 stability and expression to promote OA progression by up-regulating ADAMTS12 expression.

**Supplementary Information:**

The online version contains supplementary material available at 10.1186/s10020-023-00661-2.

## Introduction

Osteoarthritis (OA) is a main cause of disability and is a degenerative joint disease that affects > 242 million individuals worldwide (Ghouri and Conaghan 2021). The prevalence of OA is increasing due to the ageing population and some risk factors such as obesity and inflammation (Ghouri and Conaghan 2021). Chondrocyte dysfunction leads to chondrocyte extracellular matrix degradation and osteoarthritis (Ramasamy et al. 2021). Hence, attenuating cartilage/chondrocyte damage might help improve OA. Some molecular biomarkers have been regarded as potential therapeutic targets for OA, such as insulin-like growth factor 1, transforming growth factor‑β, and a disintegrin and metalloproteinase with thrombospondin motifs 5 (ADAMTS5) (Wen et al. 2021; Yoo et al. 2022; Jiang et al. 2021). However, more biomarkers should be studied to further understand the pathological mechanisms of OA.

Proteins belonging to the ADAMTS family are expressed in the cartilage and are associated with joint health and diseases, including OA (Yang et al. 2017). Among them, ADAMTS4, ADAMTS5, and ADAMTS7 have been reported to act as potential targets for OA treatment (Jiang et al. 2021; Verma and Dalal 2011; Zhang et al. 2015), and ADAMTS12 is an important target for cancer, diabetes mellitus, and stroke treatment (Li et al. 2020a; Tastemur et al. 2021; Witten et al. 2020). Researchers have revealed that ADAMTS12 is required for the inflammatory response (Moncada-Pazos et al. 2018) and is associated with cartilage oligomeric matrix protein degradation (Luan et al. 2008), suggesting the role of ADAMTS12 as a promising target for OA treatment.

Signal transducer and activator of transcription 1 (STAT1) is a nuclear transcription factor that regulates genes associated with cell survival and inflammatory response (Butturini et al. 2020). STAT1 is involved in infection, immunity, and inflammation (Mogensen and Transcription Factors 2018; Benedetti et al. 2021). Studies have reported that STAT1 expression was increased in inflammatory arthritis and lipopolysaccharides (LPS)-induced OA model, and STAT1 suppression attenuated LPS-induced inflammation (Walker et al. 2006; Jin et al. 2021) and pyroptosis (Xu and Xu. 2021) in chondrocytes. We highly suspected that the abnormal ADAMTS12 elevation in OA might be related to the transcriptional activity of STAT1 on it by analyzing the promoter region sequence of ADAMTS12.

N6-methyladenosine (m6A) modification regulates stability of gene expression to drive OA progression (Chen et al. 2021). Methyltransferase-like 3 (METTL3) is an m6A writer that regulates m6A levels by acting as the major methyltransferase, which is involved in OA progression by regulating extracellular matrix (ECM) degradation, inflammatory response, and chondrocyte damage (Sang et al. 2021; (Liu et al. 2019a). Insulin-like growth factor 2 mRNA-binding protein 2 (IGF2BP2) acts as the m6A reader to recognize m6A and stabilize m6A-modified mRNAs (Bi et al. 2019) and is suggested to be involved in the bone function and inflammatory response (Liu et al. 2018; Wang et al. 2021). Previous studies revealed that m6A of STAT1 mRNA is mediated by METTL3 (Liu et al. 2019b), but the modification mechanism of STAT1 m6A in OA remains unknown so far. Thus, this study aimed to explore the involvement of the m6A writing and reading process mediated by METTL3 and IGF2BP2 in the expressional regulation of STAT1, thereby affecting ADAMTS12 in OA progression.

This study established rat and cell OA models to clarify the molecular mechanism of ADAMTS12 overexpression in OA and demonstrated the effect of METTL3/IGF2BP2-mediated m6A modification on the STAT1/ADAMTS12 regulatory axis. This study may propose a novel understanding of OA pathogenesis and provide new targets for OA treatment.

## Materials and methods

### Patient sample collection

OA cartilage specimens with visible lesions were obtained from five patients who were diagnosed with severe hip OA and had total hip replacement surgery (Age: 71.8 ± 3.1; Female: 3; Male: 2). Specimens of normal cartilage were obtained from five patients who had total hip replacement surgery due to a fresh traumatic femoral neck fracture (Age: 73.2 ± 6.1; Female: 3; Male: 2). No hip disease had been diagnosed in the medical history of patients with a traumatic femoral neck fracture, and macroscopic examination confirmed their intact and smooth cartilage tissues. All surgeries were performed by the same team of orthopedists. This study was approved by the Medical Ethics Committee of Hunan Provincial People’s Hospital (the first affiliated hospital of Hunan Normal University), [2022] Scientific Research Ethics Review NO: [120], and the patients were between 65 and 85 years of age with informed consent. Relative experiments were repeated 5 times in patients.

### Animal experiments

The Sprague-Dawley male rats (12 weeks old, 300–350 g) obtained from Jiangsu Aniphe Biolaboratory Inc. (Nanjing, China) were acclimated for 1 week at 23℃ ± 1℃ with a 12-h light/dark cycles. The rats were randomly divided into 3 groups (n = 5/group): sham, anterior cruciate ligament transection (ACL-T), and ACL-T + S-Adenosylhomocysteine (SAH). ACL transection surgery was performed in the ACL-T group to induce the OA model following a previous study (Ma et al. 2020). Rats in ACL-T + SAH group underwent ACL-T transection surgery and were injected with 10 mg/kg of METTL3 inhibitor SAH (SAH; MedChemExpress, Monmouth Junction, NJ, USA) dissolved in normal saline into the right knee joint. Rats were euthanized with 5% isoflurane and cervical dislocation after 4 weeks. The cartilage tissues were collected and used for related analyses. This study was approved by Institutional Animal Care and Use Committee of Hubei Provincial Academy of Preventive Medicine with grant No.202120223.

### Histopathological analysis

The cartilage damage was investigated by Periodic Acid-Schiff (PAS), hematoxylin-eosin (HE), and safranin O-fast green assays referring to previous studies (Wu et al. 2015; (Li et al. 2020b; Chang et al. 2021; Liao et al. 2021). In brief, cartilage tissues were fixed in 4% paraformaldehyde (Beyotime, Shanghai, China), and then decalcified by immersion in a decalcification solution for softening, decalcified in 10% disodium ethylenediaminetetraacetic acid (EDTA; Solarbio) until complete demineralization, embedded in paraffin, and cut into 3-µm tissue sections, followed by staining with PAS (Beyotime), HE (Beyotime), and safranin O-fast green (Solarbio) following the manufacturer’s instructions. The stained sections were observed using a 200 × magnification microscope (Olympus, Tokyo, Japan). The cartilage structure damage was investigated using the Osteoarthritis Research Society International (OARSI) score referring to a previous report (Pritzker et al. 2006).

### Micro-computed tomography (CT) assay

The cartilage tissues were fixed in 4% paraformaldehyde and then scanned by micro-CT for morphological evaluation of the cartilage damage.

### Immunohistochemistry (IHC) staining

The 3-µm tissue sections completely decalcified by 10% EDTA were incubated with 3% bovine serum albumin (Solarbio) and then incubated overnight with antibodies against STAT1 (ab230428, 1:100 dilution, Abcam, Cambridge, MA, USA), ADAMTS12 (24934-1-AP, 1:100 dilution, ProteinTech, Wuhan, China), and METTL3 (ab221795, 1:300 dilution, Abcam), followed by incubating with immunoglobulin G (IgG) (ab205718, 1:5000, Abcam) conjugated by horseradish peroxidase (HRP) for 20 min. Then, the sections were reacted with a 3,3’-diaminobenzidine kit (Beyotime) and re-stained with hematoxylin (Beyotime), followed by observation under a 200× magnification microscope.

### Chondrocyte isolation and treatment

The primary chondrocytes were isolated from knee articular cartilage tissues of rats using trypsin and collagenase II following a previous report (Zhang et al. 2022). Chondrocytes were cultured in DMEM/F12 medium (Gibco, Grand Island, NY, USA) containing 10% fetal bovine serum (Gibco) and 1% penicillin/streptomycin (Gibco) at 37 °C in 5% CO_2_. Interleukin-1 beta (IL-1β) was used to mimic the inflammatory environment in cultured chondrocytes. This study incubated chondrocytes with 10 ng/mL of IL-1β (MedChemExpress) to induce OA cellular model as previously reported (Zhang et al. 2022). Relative experiments were repeated 3 times in rat primary chondrocytes.

### Cell transfection

The overexpression vectors (OE-METTL3, OE-ADAMTS12, or OE-STAT1) and empty vector (OE-NC), shRNAs (sh-STAT1, sh-METTL3 or sh-IGF2BP2), and negative control (NC) were provided via Genscript (Nanjing, China). Chondrocytes were transfected with vectors or shRNAs using the Xfect™ RNA Transfection Reagent (Takara, Dalian, China). Cells were collected for IL-1β stimulation 24 h post-transfection.

### Quantitative polymerase chain reaction (qPCR)

Total RNAs were isolated using TRIzol reagent (Vazyme, Nanjing, China), and 500 ng of RNA samples were used to synthesize cDNA using a Reverse Transcription kit (Promega, Madison, WI, USA), followed by qPCR with a ReverTra Ace qPCR RT Kit (Toyobo, Tokyo, Japan). The primer sequences were synthesized by Genscript (Nanjing, China), and listed as ADAMTS12: (Forward, 5’-CCATGTGAAGATGGCGGCT-3’; Reverse, 5’-ATCCAGTCAGTCCTTGGCAG-3’), STAT1 (Forward, 5’-CGATTTAATCAGGCCCAGGAGG-3’; Reverse, 5’-TGCTCTATGCACATGACTTGGTC-3’), METTL3 (Forward, 5’- CAGAGCAAGAAGGTCAGTCAGG-3’; Reverse, 5’-CTCTTCCTTGGTCCCATAGTCAC-3’), and GAPDH (Forward, 5’-GCAAGTTCAACGGCACAG-3’; Reverse, 5’-GCCAGTAGACTCCACGACAT-3’). GAPDH was regarded as a control, and relative RNA expression was calculated referring to the 2^−ΔΔCt^ method.

### Chromatin immunoprecipitation (ChIP)-qPCR

The lysed cartilages or chondrocytes were fixed with 1% formaldehyde and then quenched with glycine. The chromatin lysates were obtained by ultrasonic with Sonifier (Branson, Missouri, MO, USA) to obtain DNA fragments of 200–1000 bp. ChIP assay was performed using a SimpleChIP kit (Cell Signaling, Danvers, MA, USA) following the manufacturer’s instructions. The antibodies anti-STAT1 (LS-B591, 1:100 dilution, LifeSpan BioSciences, Seattle, WA, USA) or IgG were used for DNA sample immunoprecipitation. ADAMTS12 DNA level was detected by qPCR.

### Electromobility shift assay (EMSA)

The oligonucleotide probes of ADAMTS12 were synthesized by Sangon (Shanghai, China) according to STAT1 sites of the ADAMTS12 promoter. Cartilage samples were homogenized and the nuclear pellet was collected for EMSA assay using a LightShift Chemiluminescent EMSA Kit (Thermo Fisher Scientific) following the manufacturer’s protocols (Li et al. 2021). The following oligonucleotide probes were used: wild type (WT)-labeled probe (5’-GGTGATGAGGGTAGTACCCGGCGTTCTTGGAAACGCAGAGGAGGAGGAAAA TAGAAGCGG-3’), WT competitor probe (5’-GGTGATGAGGGTAGTACCCGGCGTT CTTGGAAACGCAGAGGAGGAGGAAAATAGAAGCGG-3’), and mutant (MUT) competitor probe (5’-GGTGATGAGGGTAGTACCCGAATGATACACGAAAAGTGCGGAGGAGGAA AATAG AAGCGG-3’).

### Western blot

Proteins were isolated using a radioimmunoprecipitation assay lysis buffer (Beyotime), and concentrations were determined using a bicinchoninic acid kit (Thermo Fisher Scientific). Protein samples (20 µg) were electrophoretically separated by sodium dodecyl sulfate-polyacrylamide gel electrophoresis and were electro-transferred on polyvinylidene fluoride membranes (Bio-Rad, Hercules, CA, USA), followed by immersing in 5% non-fat milk. The membranes were incubated overnight using primary antibodies against ADAMTS12 (24934-1-AP, 1:100 dilution, ProteinTech), STAT1 (LS-B591, 1:1000 dilution, LifeSpan BioSciences), METTL3 (ab221795, 1:1000 dilution, Abcam), or GAPDH (ab9485, 1:3000, Abcam) after blocking the non-specific sites, and then incubated with HRP-conjugated IgG (ab205718, 1:20000, Abcam) for 2 h, followed by ECL kit (Thermo Fisher Scientific) exposure. GAPDH and β-actin were considered as references.

### Dual-luciferase reporter assay

The WT or MUT sequences of ADAMTS12 promoter were inserted in psiCheck2 vectors (Promega) to generate the reporter vectors, and co-transfected with pcDNA3.1 empty vector (OE-NC) or STAT1 overexpression vector (OE-STAT1) in chondrocytes. Luciferase activity was detected using a dual-luciferase reporter assay system (Promega) after 48 h. Relative experiment were repeated 3 times in 293T cells.

### RNA immunoprecipitation (RIP)

RIP assay was performed by adopting the Magna RIP kit (Sigma-Aldrich) following the manufacturer’s protocols. The cartilage samples from patients or rats and chondrocytes were lysed and incubated with magnetic beads conjugated via anti-IGF2BP2 (11601-1-AP, 1:100 dilution, PeorteinTech) or IgG (ab205718, 1:200 dilution, Abcam) overnight. The immunoprecipitated mSTAT1 was examined and expressed as % of input (cell lysates).

### MeRIP-qPCR

The mRNAs from cartilage samples or chondrocytes were incubated with anti-m6A-conjugated magnetic beads with a Magna MeRIP™ m6A kit (Sigma-Aldrich) following the manufacturer’s protocols. The m6A-modified STAT1 was immunoprecipitated and detected by qPCR.

### Actinomycin D assay

The treated chondrocytes (2 × 10^5^/well) were added to 6-well plates and stimulated with 2 µg/mL of actinomycin D (Selleck, Shanghai, China) for 0, 2, 4, 6, 8, or 10 h. Then, chondrocytes were lysed, and the STAT1 mRNA remaining level was examined by qPCR.

### Immunofluorescence (IF) staining

IF staining was performed following the manufacturer’s protocols of a previous report (Ke et al. 2022). In brief, the treated chondrocytes (2 × 10^5^/well) were placed on the coverslip in 6-well plates and then fixed using 4% paraformaldehyde. Then, chondrocytes were treated with 1% Triton X-100 and 3% H_2_O_2_, followed by 5% bovine serum albumin immersion. Next, cells were incubated overnight with primary antibodies against METTL3 (ab221795, 1:100 dilution, Abcam), STAT1 (LS-B591, 1:50 dilution, LifeSpan BioSciences), ADAMTS12 (NBP2-68996, 1:100 dilution, Novus Biologicals, Littleton, CO, USA), and fluorescent secondary antibodies (GB21303, 1:500 dilution, Servicebio) for 1 h. The nuclei were labeled by incubating with 4’,6-diamidino-2-phenylindole (DAPI). Cells were observed under a fluorescence microscope (Keyence, Osaka, Japan).

### Apoptotic ratio by flow cytometry analysis

Cell samples were collected from each group, including those floating in the medium. The cell samples to be tested were obtained after cleaning, centrifugation, and Binding Buffer resuspension. Annexin V-FITC at 5 µL was added and incubated at room temperature for 15 min in the dark, followed by 5 µL of propidium iodide, before testing. Flow cytometry (CytoFLEX, BECKMAN) was used for detection.

### Apoptosis observed by TdT - mediated dUTP Nick-End labeling (TUNEL)

Cells were fixed with 4% paraformaldehyde for 20 min, rinsed in phosphate-buffered saline (PBS), and treated with ethanol/acetic acid (2:1) at 20 °C for 5 min. Then, cells were washed with PBS and permeated at room temperature for 15 min with 0.2% Triton X-100 diluted in 0.1% sodium citrate (w/v). Then, cells were immersed for 30 min in TUNEL buffer: 30 mM of Tris-HCl buffer (pH of 7.2), 140 mM of sodium cacodylate, 1 mM of cobalt chloride, and 0.3% Triton X-100. The cells were washed with PBS after incubating for 2 h at 37 °C in the TUNEL reaction mixture (Roche Diagnostics), and then incubated at room temperature in the dark with Cy3-conjugated streptavidin (1:500; Jackson ImmunoResearch Laboratories), and then counterstained with 1:2,000 DAPI.

### Statistical analysis

Data are presented as mean ± standard deviation from three independent experiments. The difference was analyzed by student’s t-test or Analysis of Variance followed by the least significant difference test using Statistical Package for the Social Sciences version 17.0 (SPSS, Chicago, IL, USA). The difference was considered significant at *P* < 0.05.

## Results

### The up-regulated ADAMTS12 in OA is related to the transcriptional activation of STAT1

**Fig. 1:**
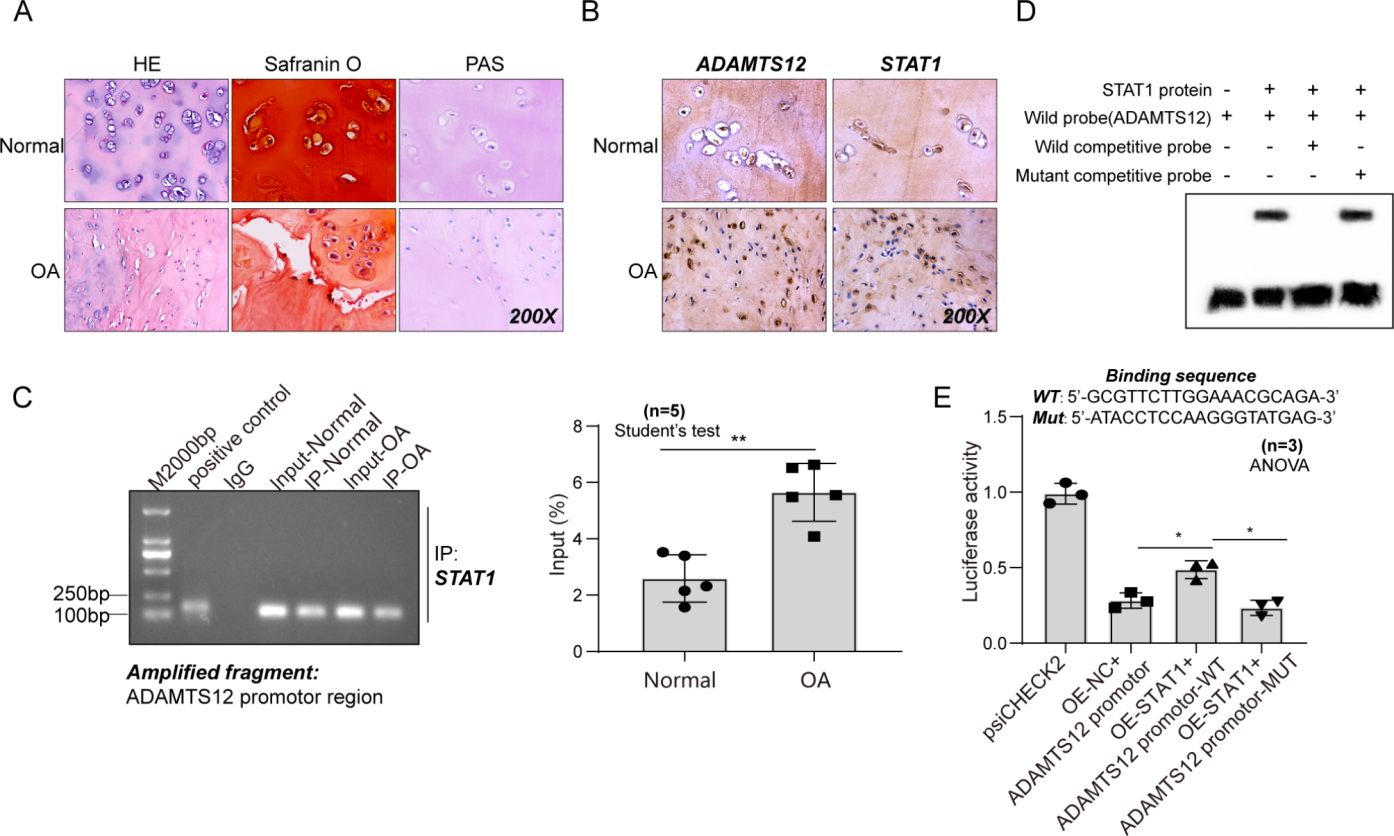
STAT1 regulates ADAMTS12 transcription. Tissues of patients with OA and cartilage tissues of controls. (**A**) HE, PAS, and safranin O-fast green of human cartilage tissues. n = 5 (**B**) IHC analysis for STAT1 and ADAMTS12 levels in human cartilage tissues. n = 5 (**C** and **D**) The binding of STAT1 on ADAMTS12 promoter in human cartilage tissues by ChIP-qPCR and EMSA analyses. n = 5 (**E**) Luciferase reporter assay for binding of STAT1 on ADAMTS12 promoter in 293T cells. n = 3. ^*^P < 0.05; **, *P* < 0.01

Obvious histological changes were observed in the cartilage of patients with OA (Fig. 1A). The safranin O-fast green and PAS staining suggested a significant reduction in the content of cartilage. The protein expression levels of ADAMTS12 and STAT1 measured by the IHC staining in cartilaginous tissue of pathological changes were significantly increased compared with the non-pathological ones (Fig. 1B). Further, STAT1 was confirmed to bind to the promoter region of ADAMTS12 through the ChIP-qPCR assay in cartilage samples (Fig. 1C). Additionally, STAT1 over-expression increased the transcriptional activity of ADAMTS12 by binding motifs of 5’-GCGTTCTTGGAAACGCAGA-3’ through the EMSA and dual-luciferase reporter analysis (Fig. 1D–E).

**Fig. 2:**
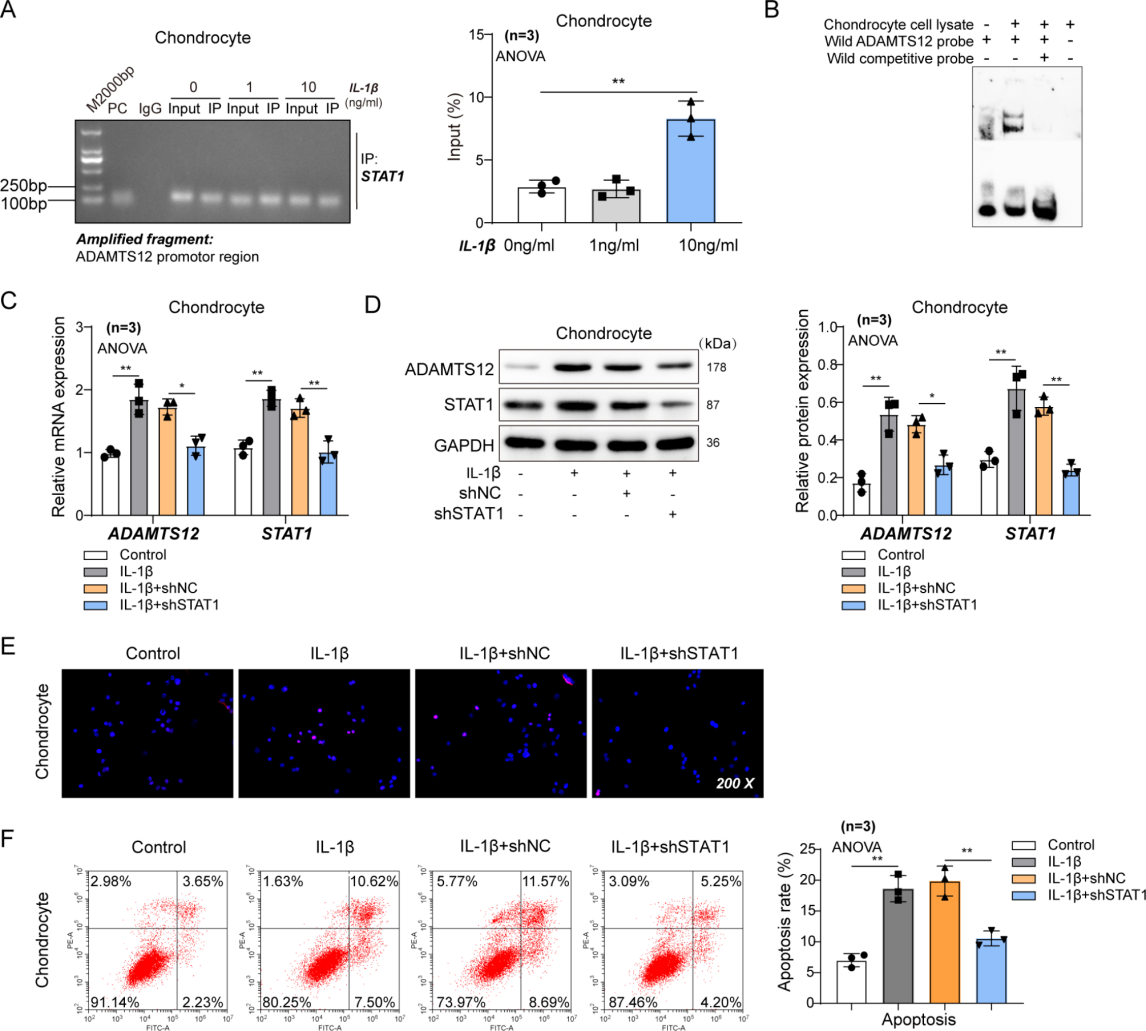
STAT1 regulates ADAMTS12 transcription. Rat chondrocytes were transfected with NC or shSTAT1, followed by IL-1β incubation. Cells were grouped as control, IL-1β, NC + IL-1β, or shSTAT1 + IL-1β. (**A**) ChIP-qPCR analysis for binding of STAT1 on ADAMTS12 promoter in rat chondrocytes stimulated with or without IL-1β. n = 3 (**B**) The binding of STAT1 on ADAMTS12 promoter in rat chondrocytes through EMSA assay. n = 3 (**C**-**D**) RT-qPCR and western blot assays for STAT1 and ADAMTS12 levels in rat chondrocytes. n = 3 (**E**) TUNEL staining of rat chondrocyte. n = 3 (**F**) Apoptosis of rat chondrocyte detected by flow cytometry. n = 3. ^*^P < 0.05; **, *P* < 0.01

Next, STAT1 binding to the ADAMTS12 promoter region was found in a model of IL-1β -induced chondrocyte inflammation (Fig. 2A–B). Knocking down STAT1 by shRNA in the presence of IL-1β (Figure S1) resulted in a significant decrease in the mRNA and protein levels of ADAMTS12 and STAT1 (Fig. 2C–D). Meanwhile, the apoptosis of chondrocytes was significantly reduced when shRNA interfered with STAT1 (Fig. 2E–F).

These results prove that STAT1 promotes ADAMTS12 expression in a transcriptional regulation manner under the inflammatory environment, leading to the chondrocyte extracellular matrix degradation.

**Fig. 3:**
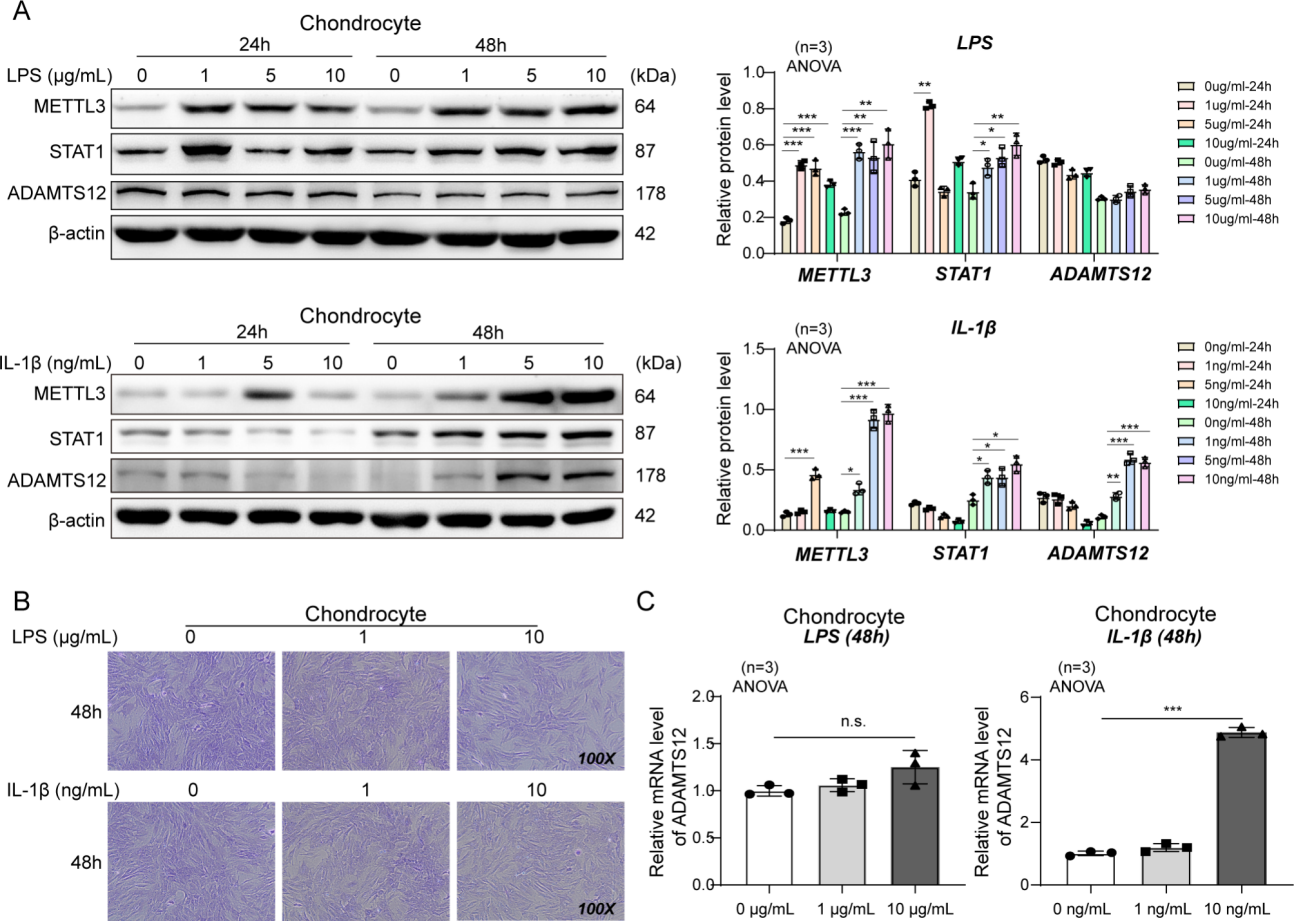
IL-1β induced the increased expression of ADAMTS12. Rat chondrocytes were treated with different concentrations of LPS or IL-1β for 24 or 48 h. (**A**) Relative protein expression tested by western blotting. n = 3 (**B**) Toluidine blue staining of rat chondrocytes. n = 3 (**C**) Relative mRNA expression tested by RT-qPCR. n = 3. ^*^P < 0.05; **, *P* < 0.01; ***, *P* < 0.001

In particular, chondrocyte inflammation is induced by exposing chondrocytes to a culture medium containing LPS or IL-1β. However, LPS could not increase the mRNA or protein expression levels of ADAMTS12, while IL-1β could (Fig. 3). Therefore, IL-1β was chosen as an inductor of chondrocyte inflammation throughout the study.

### METTL3 mediates m6A modification of STAT1 in OA

**Fig. 4:**
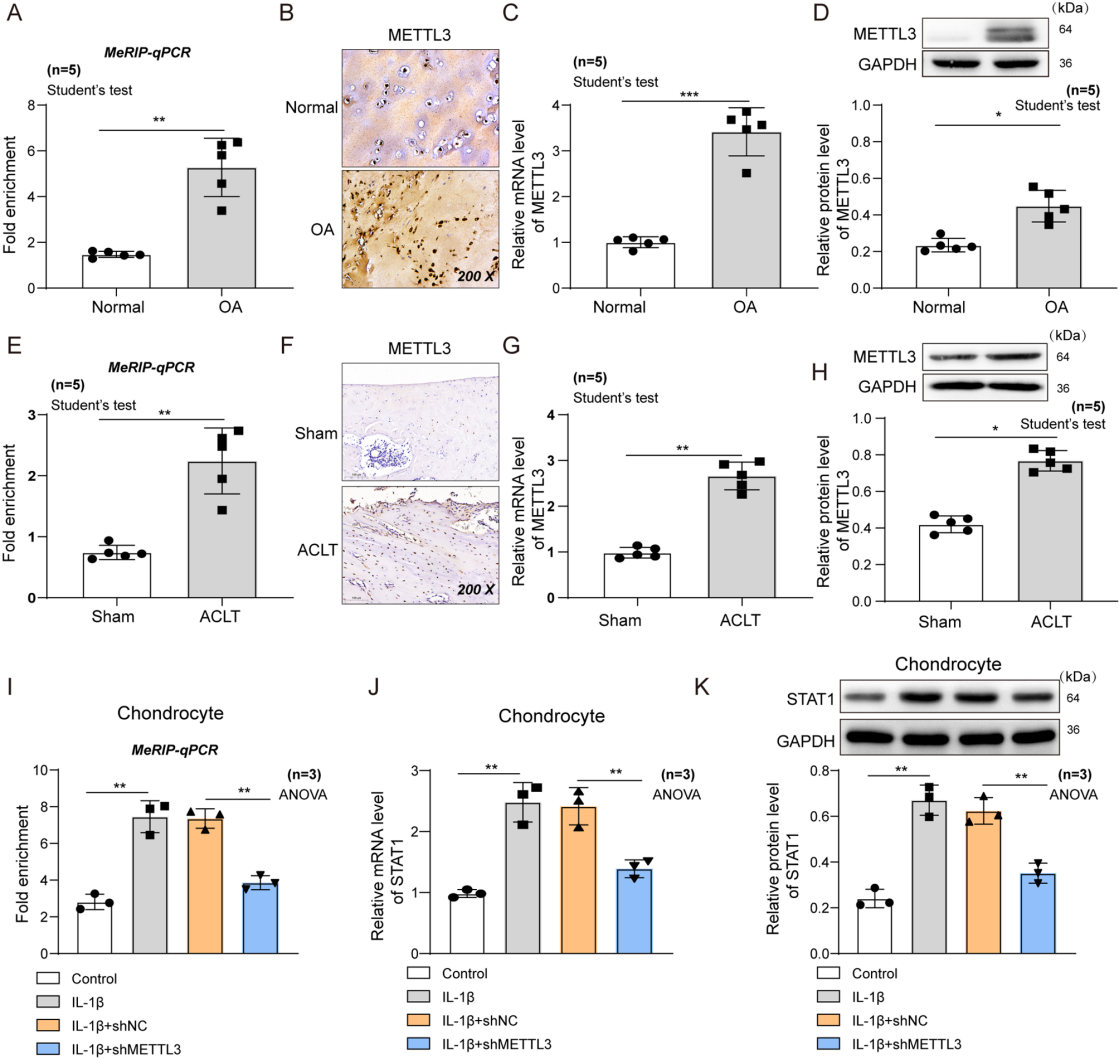
METTL3 regulates STAT1 expression. (**A**) MeRIP-qPCR assay for m6A methylation level of STAT1 in cartilage tissues of normal cases or patients with OA. n = 5. (**B**–**D**) METTL3 levels in cartilage tissues of normal or patients with OA by IHC, RT-qRCR, and western blot assays. n = 5. (**E**) MeRIP-qPCR assay for m6A methylation level of STAT1 in cartilage tissues in sham or OA rat model groups. n = 5. (**F**–**H**) METTL3 levels in cartilage tissues in sham or OA rat model groups by IHC, RT-qRCR, and western blot. n = 5. (**I**) MeRIP-qPCR assay for m6A methylation level of STAT1 in rat chondrocytes transfected with NC or shMETTL3 before IL-1β stimulation. n = 3. (**J**–**K**) The mRNA and protein expression levels of STAT1 in rat chondrocytes by qPCR and western blot assays. n = 3. ^*^P < 0.05; **, *P* < 0.01; ***, *P* < 0.001

The RNA methylation level of STAT1 was analyzed in cartilage tissues to clarify why STAT1 is up-regulated in OA and then affected ADAMTS12 expression. Rat OA models established by ACL-T with significant cartilage pathological changes and increased ADAMTS12 expression were presented (Figure S2). The result revealed a noticeably higher methylation level in the cartilage tissues of patients with OA than in normal cases (Fig. 4A). The methyltransferase METTL3 expression, as an m6A writer, was significantly enhanced in cartilage tissues of patients with OA compared to the normal group (Fig. 4B–D), consistent with that in OA rat models (Fig. 4E–H). Additionally, the m6A modification degree of STAT1 was detected and was enhanced undergoing IL-1β stimulation in the chondrocyte inflammation cells, whereas METTL3 silencing by shRNA (Figure S3) attenuated this effect (Fig. 4I). Similarly, the mRNA and protein levels of STAT1 revealed the same changes to its m6A modification levels in each group (Fig. 4J–K). These results indicates that METTL3 up-regulates STAT1 expression by enhancing its m6A modification.

### IGF2BP2 mediates mSTAT1 stabilization and promotes its expression in OA

**Fig. 5:**
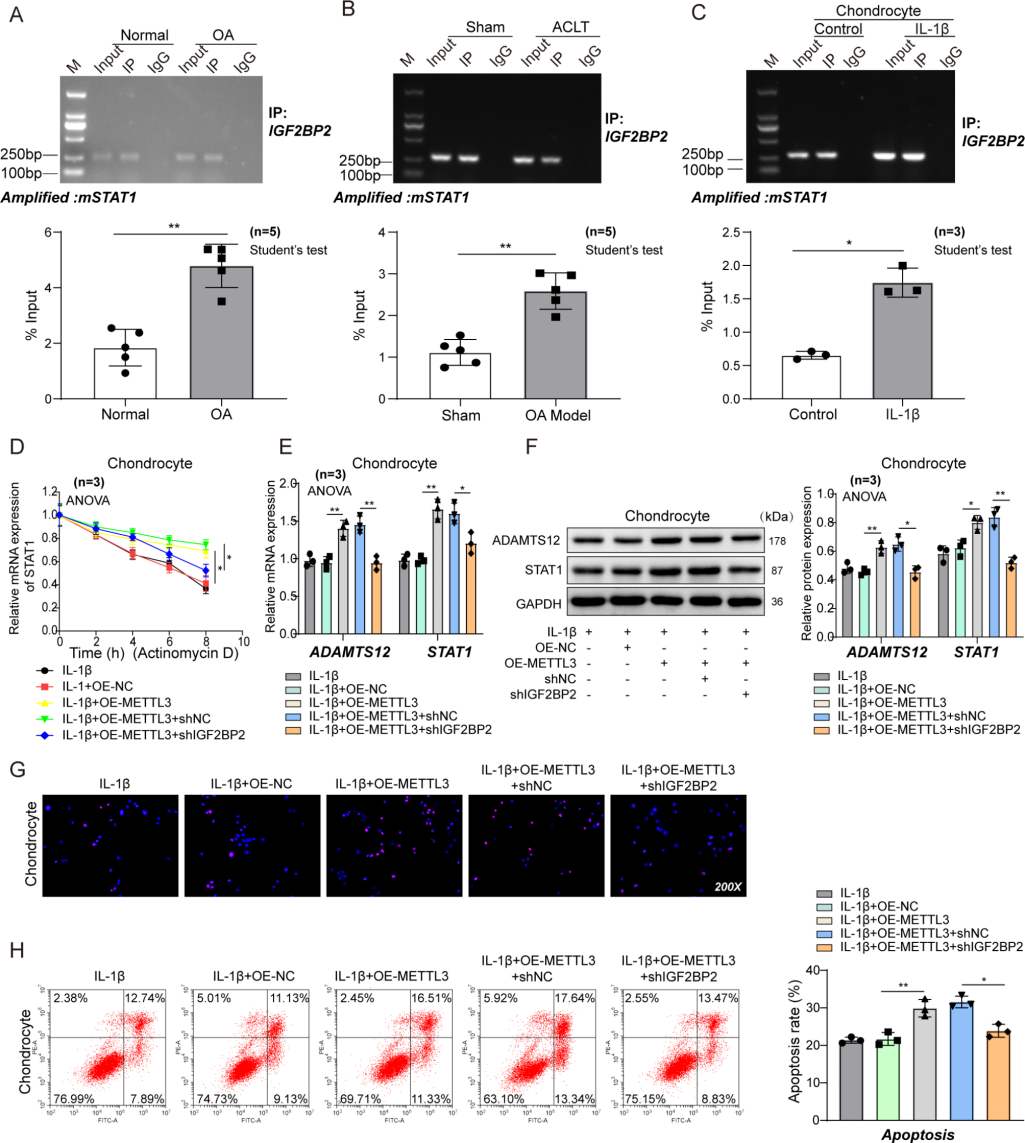
METTL3/IGF2BP2 axis regulates STAT1 stability and expression. (**A**–**B**) RIP assay for binding of IGF2BP2 and STAT1 in patients with OA and rat models. n = 5. (**C**) RIP assay for binding of IGF2BP2 and STAT1 in IL-1β-treated rat chondrocytes. n = 3. (**D**) STAT1 stability detected via RT-qPCR in IL-1β-treated rat chondrocytes transfected with NC or shIGF2BP2 through actinomycin D assay. n = 3. (**E**-**F**) The mRNA and protein levels of STAT1 and ADAMTS12 in IL-1β-treated rat chondrocytes transfected with OE-NC, OE-METTL3, OE-METTL3 + NC, or OE-METTL3 + shIGF2BP2 by RT-qPCR and western blot. n = 3. (**G**) TUNEL staining of rat chondrocyte. n = 3 (**H**) Apoptosis of rat chondrocyte detected by flow cytometry. n = 3. ^*^P < 0.05; **, *P* < 0.01; ***, *P* < 0.001

M6A-modified RNAs may be degraded or more stable, thereby showing different translation levels. IGF2BP2 is known as the m6A reading protein and is an RNA-binding protein that recognizes the methylation modifications on mRNA and promotes RNA stabilization. The RIP-qPCR assay in cartilage tissues both from clinic and rat models presented that IGF2BP2 could bind with mSTAT1, especially in patients with OA and rats of OA model (Fig. 5A–B). Similarly, the binding relationship between IGF2BP2 and mSTAT1, as well as the mRNA expression levels of mSTAT1, in chondrocyte inflammatory cells were enhanced after IL-1β treatment by fold change expression (Fig. 5C), which suggests that IGF2BP2 may only recognize and bind to m6A modified mSTAT1, thereby up-regulating its expression.

We performed RNA resistance tests in chondrocytes over-expressing METTL3 or simultaneously knocking down IGF2BP2 to determine whether the up-regulation of mSTAT1 was due to the elevation of m6A modification. The results revealed more amplified mSTAT1 through qPCR detection in the METTL3 over-expressed group. However, the abundance of mSTAT1 significantly decreased when shRNA interfered with IGF2BP2 expression (Fig. 5D). These cell samples were tested before and after IL-1β induction to evaluate the effects of the simulated inflammatory environment on m6A modification and mSTAT1 expression level. Additionally, the expression levels of STAT1 and ADAMTS12 up-regulated in IL-1β-treated chondrocytes were augmented by METTL3 over-expression, and this effect was reversed because of IGF2BP2 silencing (Fig. 5E–F). Concurrently, the increased apoptosis of chondrocyte cells induced by METTL3 up-regulation could be restored after IGF2BP2 interference (Fig. 5G–H). These results presented that METTL3/IGF2BP2 axis enhanced STAT1 stability and expression in OA.

### METTL3 promotes chondrocyte injury by up-regulating ADAMTS12 in vitro and in vivo

**Fig. 6:**
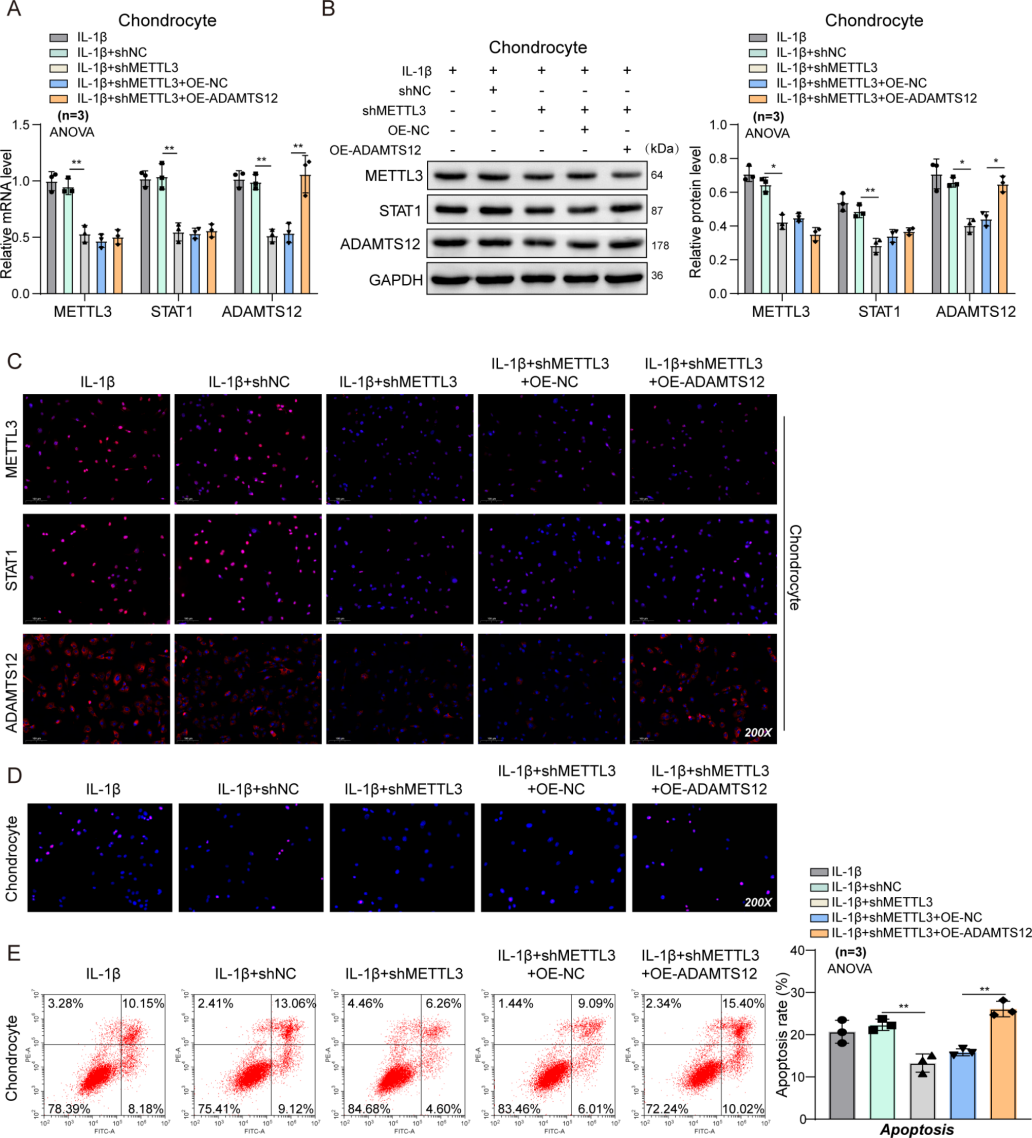
METTL3 regulates IL-1β-treated chondrocytes by modulating ADAMTS12. Rat chondrocytes were transfected with NC, shMETTL3, shMETTL3 + OE-NC, or shMETTL3 + OE-ADAMTS12 followed by IL-1β stimulation. (**A**-**B**) RT-qPCR and western blot for METTL3 and ADAMTS12 expressions in IL-1β-treated rat chondrocytes. n = 3. (**C**) IF assays for METTL3, STAT1, and ADAMTS12 levels in treated rat chondrocytes. n = 3 (**D**) TUNEL staining of rat chondrocyte. n = 3 (**E**) Apoptosis of rat chondrocyte detected by flow cytometry. n = 3. ^*^P < 0.05; **, *P* < 0.01; ***, *P* < 0.001

In vivo and in vitro experiments were conducted to investigate whether METTL3 could regulate ADAMTS12 expression by affecting STAT1 in OA. Relative mRNA and protein levels of METTL3, STAT1, and ADAMTS12 were increased after IL-1β stimulation, which was then reversed by reducing METTL3 through shRNA. Transfection with ADAMTS12 over-expression plasmid increased ADAMTS12, but it did not change the METTL3 levels (Fig. 6A–C). Additionally, the apoptosis in IL-1β-treated chondrocytes was decreased by METTL3 silencing, which could be reversed by ADAMTS12 over-expression (Fig. 6D–E). These data support our belief that the effects of METTL3 on ADAMTS12 in OA depend on the transcriptional activity of STAT1. The functional acquisition cell model of METTL3 and ADAMTS12 were shown in Figures S3 and S4.

**Fig. 7:**
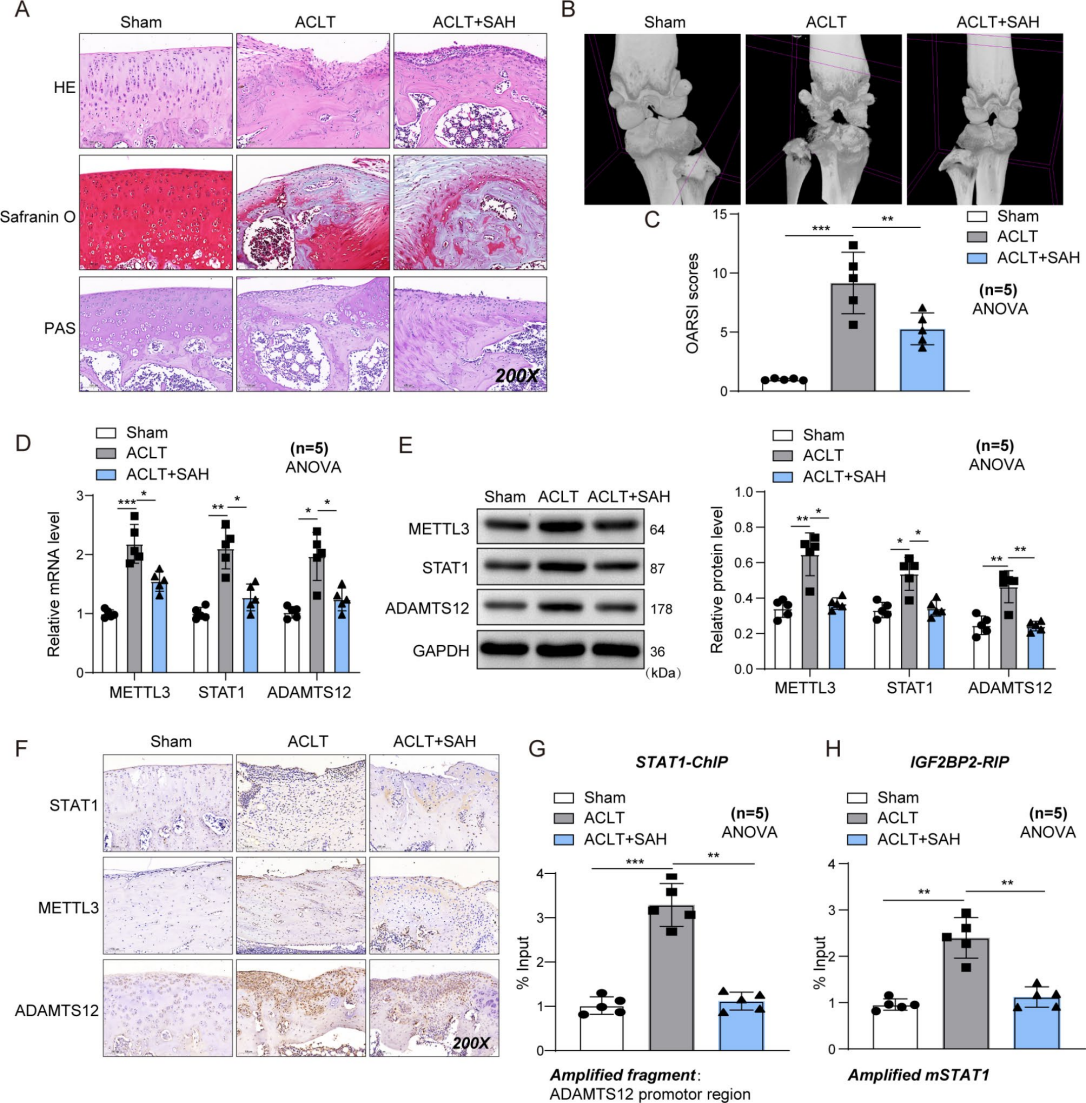
METTL3 regulates osteoarthritis progression in OA rats via mediating ADAMTS12. OA rats were induced by the ACL-T method and then treated with METTL3 inhibitor SAH. Rats were grouped as sham, ACL-T, and ACL-T + SAH. (**A**) HE, PAS, and safranin O-fast green of rat cartilage tissues in each group. n = 5 (**B** and **C**) OARSI score and micro-CT assays were performed in each group. n = 5. (**D**–**F**) RT-qPCR, western blot, and IHC assays for METTL3, ADAMTS12, and STAT1 levels in rat cartilage tissues in each group. n = 5. (**G**) The binding relationship between STAT1 and ADAMTS12 promoter region in rat cartilage tissues was tested by ChIP assay. n = 5. (**H**) The binding relationship between STAT1 mRNA and IGF2BP2 in rat cartilage tissues was tested by RIP assay. n = 5. ^*^P < 0.05; **, *P* < 0.01; ***, *P* < 0.001

Subsequently, we analyzed the effects of the METTL3/ADAMTS12 axis on ACL-T-induced OA rat models. The rats were divided into the sham, ACL-T, and ACL-T + SAH (specific small molecule inhibitor of METTL3) groups. The results of HE, PAS, and safranin O-fast green revealed that the cartilage damage and fibrosis induced by ACL-T were mitigated because of METTL3 inhibitor SAH (Fig. 7A). The cartilage damage by mirco-CT and OARSI score were decreased due to SAH (Fig. 7B **and C**). Additionally, both mRNA and protein levels of STAT1, METTL3, and ADAMTS12 in cartilage tissues were increased in the ACL-T group compared to the sham group but significantly decreased when SAH was administered (Fig. 7D–F). These results suggest that taking SAH can significantly improve the symptoms of OA and inhibit the expression of STAT1 and ADAMTS12 in vivo. Additionally, we did not detect the over-enrichment of STAT1 in the ADAMTS12 promoter region in the cartilage tissue of ACL-T + SAH rats compared with the sham group. The enrichment degree decreased to no difference with the sham group after SAH administration although ACL-T could significantly increase the enrichment of STAT1 (Fig. 7G). STAT1 functioned as a transcription factor and could promote ADAMTS12 transcription in the OA model group by regulating its promoter region. The addition of SAH reduced the m6A modification of STAT1, thereby relieving the effects of STAT1 on ADAMTS12 transcription. Similarly, IGF2BP2 binding to STAT1 mRNA was enhanced in the ACL-T group but was not significantly different from that in the sham group in the cartilage tissue of rats administered SAH (Fig. 7H). In conclusion, the mechanism by which METTL3 inhibition can significantly alleviate OA is related to its participation in regulating the stability of mSTAT1, thereby affecting the expression level of ADAMTS12.

## Discussion

**Fig. 8:**
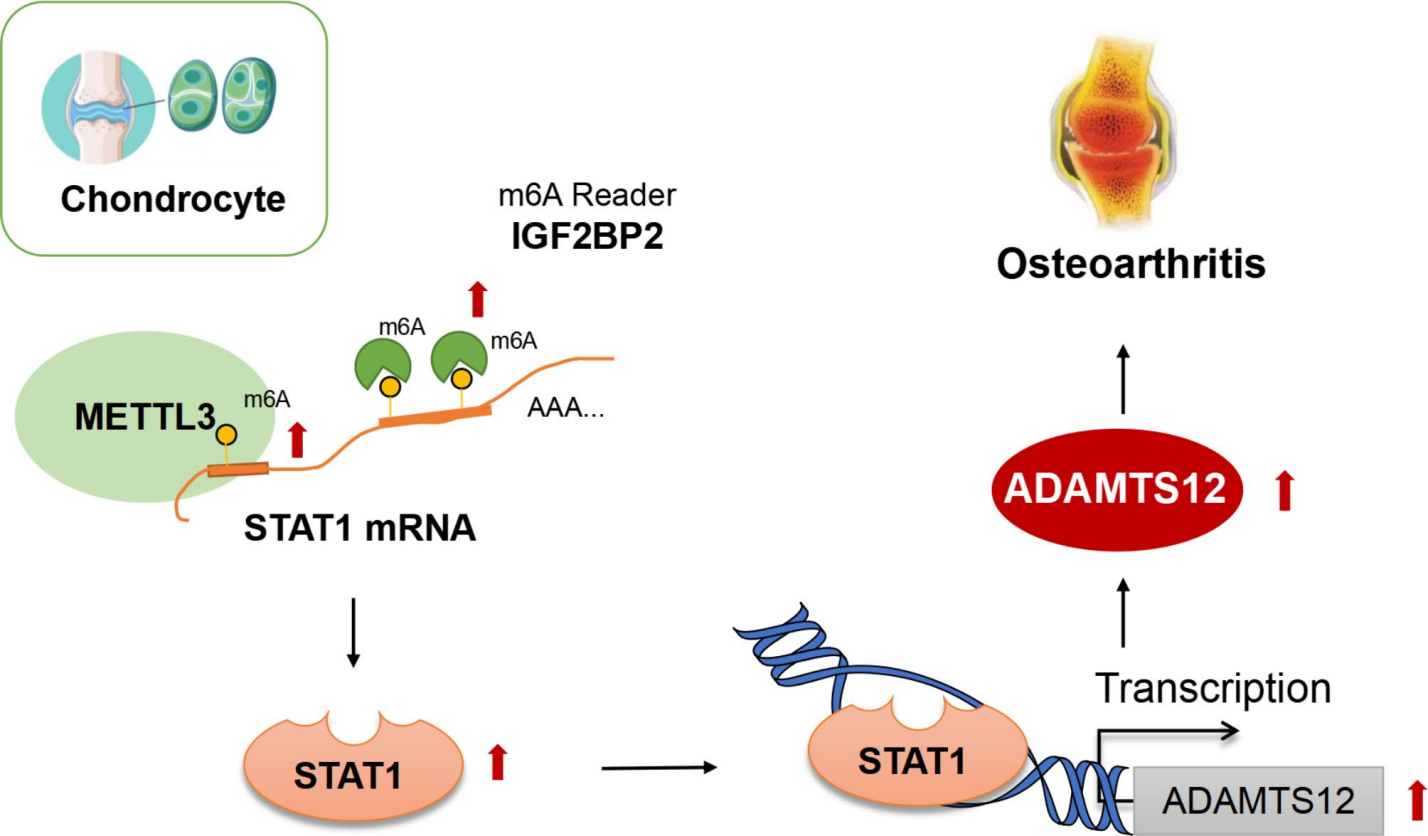
Graphic abstract of the regulation mechanism in this study

OA is a common joint disease associated with the loss of articular cartilage, which affects > 10% of people over the age of 60 years (Panikkar et al. 2021). This reduced the quality of life and elevated the morbidity of patients. Hence, we aimed to explore new targets to improve OA. This study first revealed STAT1 could transcriptionally activate ADAMTS12 in OA by establishing animal and cellular models of OA. Moreover, we confirmed that METTL3 formed the majority of m6A deposition on STAT1, and IGF2BP2 was further bound with m6A-modified regions of STAT1 to increase its stability. STAT1 was transferred to the nucleus in the presence of IL-1β to promote ADAMTS12 transcription and expression, which contributed to OA development (Fig. 8). Our research indicated new therapeutic targets of OA.

ADAMTS12 is a multifunctional metalloproteinase with important roles in inflammation (Wei et al. 2014). ADAMTS12 was significantly upregulated in the pathological tissues of patients with OA and was associated with ECM degradation and chondrocyte destruction (Luan et al. 2008; Ji et al. 2016; Li et al. 2022; Perez-Garcia et al. 2019; Liu et al. 2006), suggesting that ADAMTS12 may be a key target in OA treatment. This study revealed that IL-1β significantly stimulated ADAMTS12 up-regulation in chondrocytes, whereas LPS did not. We speculate that this is because LPS is a bacterial lipopolysaccharide, which has a good induction effect on inflammation caused by exogenous infections, while OA is a sterile inflammatory disease. The differential effects of IL-1β and LPS on ADAMTS12 expression reflect the disease specificity in which ADAMTS12 may be involved.

STAT1 is an inflammation-related transcriptional activator involved in infection and inflammatory diseases, including OA and coronavirus disease 2019 (Butturini et al. 2020; Rincon-Arevalo et al. 2022). Furthermore, STAT1 was reported to contribute to chondrocyte inflammation and damage (Jin et al. 2021; Xu and Xu 2021), indicating its role in OA development. Moreover, STAT1 was reported to function as a transcriptional activator to stimulate some gene expressions by binding with their promoters, such as sphingosine 1-phosphate receptor 1 and ankyrin repeat-, SH3 domain-, and proline-rich region-containing protein 2 (Xin et al. 2020; Turnquist et al. 2014). Our study first revealed that STAT1 can act as an important upstream transcriptional activator to up-regulate ADMATS12 expression, which could regulate OA progression by targeting ADAMTS12.

High amounts of m6A methylated mRNAs were presented in IL-1β-treated chondrocytes (Liu et al. 2019a). METTL3 is well-recognized as one of the most important m6A “writers”, and the “reader” IGF2BP2 is responsible for identifying methylated transcripts mediated by METTL3 (Xu and Xu 2021; Li et al. 2019). The current study demonstrated that METTL3 acts as a “writer” to increase the methylation level of STAT1 mRNA, which is subsequently recognized by the “reader” IGF2BP2 to stabilize it in chondrocytes, where inflammatory cytokines are present, thereby ultimately achieving elevated expression levels. This mechanism explains why STAT1 is up-regulated in pathological tissues of OA from the perspective of epigenetic regulation by m6A modification and indicates the importance of m6A in OA development. Here, we successfully reversed chondrocyte inflammatory injury by silencing METTL3, indicating the potential of targeting METTL3 in OA therapy, which was consistent with a previous study (Liu et al. 2019a).

## Conclusion

In conclusion, ADAMTS12 was an important target for the METTL3/IGF2BP2/STAT1 axis to regulate OA progression. Mechanically, METTL3 increased STAT1 stability in an IGF2BP2-dependent manner to upregulate ADAMTS12 transcription, thereby promoting OA progression. Our study might provide new preventive strategies for OA treatment by focusing on METTL3/IGF2BP2-mediated methylation, STAT1, and ADAMTS12.

## Electronic supplementary material

Below is the link to the electronic supplementary material.

Supplementary Material

## Data Availability

The datasets generated during and/or analysed during the current study are available from the corresponding author on reasonable request.

## Abbreviations

OA: Osteoarthritis
ADAMTS: A disintegrin and metalloproteinase with thrombospondin motifs
COMP: cartilage oligomeric matrix protein
IL-1β: interleukin-1beta
STAT1: Signal transducer and activator of transcription 1
LPS: lipopolysaccharides
m6A: N6-methyladenosine
METTL3: Methyltransferase-like 3
ECM: extracellular matrix
IGF2BP2: Insulin-like growth factor 2 mRNA-binding protein 2
ACL-T: anterior cruciate ligament transection
SAH: S-Adenosylhomocysteine
PAS: Periodic Acid-Schiff
HE: hematoxylin-eosin
OARSI: Osteoarthritis Research Society International
IHC: Immunohistochemistry
ChIP: Chromatin immunoprecipitation
qPCR: Quantitative polymerase chain reaction
EMSA: Electromobility shift assay
HRP: horseradish peroxidase
WT: wild type
MUT: mutant
RIP: RNA immunoprecipitation
IF: Immunofluorescence
DAPI: 4‘,6-diamidino-2-phenylindole
PI: propidium iodide
TUNEL: TdT - mediated dUTP Nick-End Labeling
PBS: phosphate buffered saline
SD: standard deviation
ANOVA: Analysis of Variance
LSD: least significant difference
EDTA: disodium ethylenediaminetetraacetic acid
RBP: RNA-binding protein
