# Supplementary material for: Epigenetic regulatory mechanism of ADAMTS12 expression in osteoarthritis
Source: Mol Med. 2023 Jul 3;29:86. doi: 10.1186/s10020-023-00661-2 (PMC10318776; doi:10.1186/s10020-023-00661-2)
Supplement: Supplementary file 1 — Supplementary Material [file 10020_2023_661_MOESM1_ESM.docx]

**
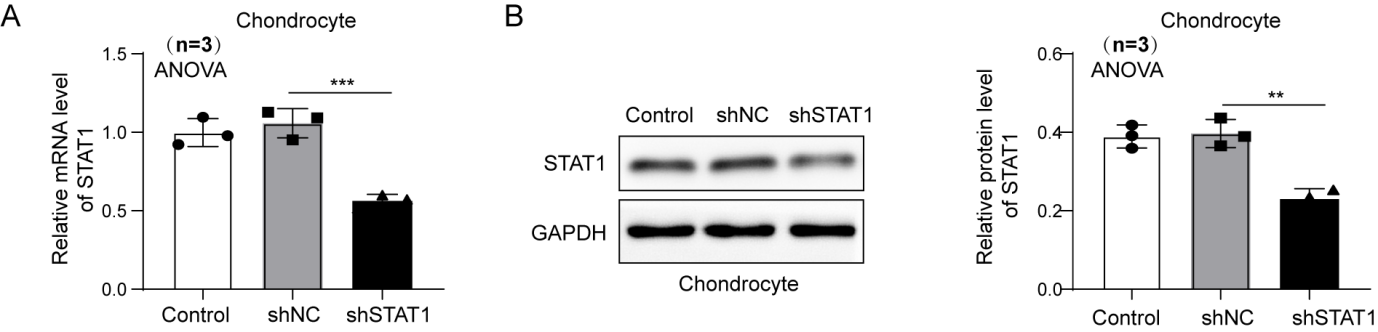
**

**Figure S1. Knockdown efficiency of shRNA on STAT1 expression in rat chondrocytes.** (A) Relative mRNA expression level of STAT1 detected by RT-qPCR. n = 3 (B) Relative protein expression level of STAT1 detected by western blot. n = 3. **, *P* < 0.01; ***, *P* < 0.001.

**
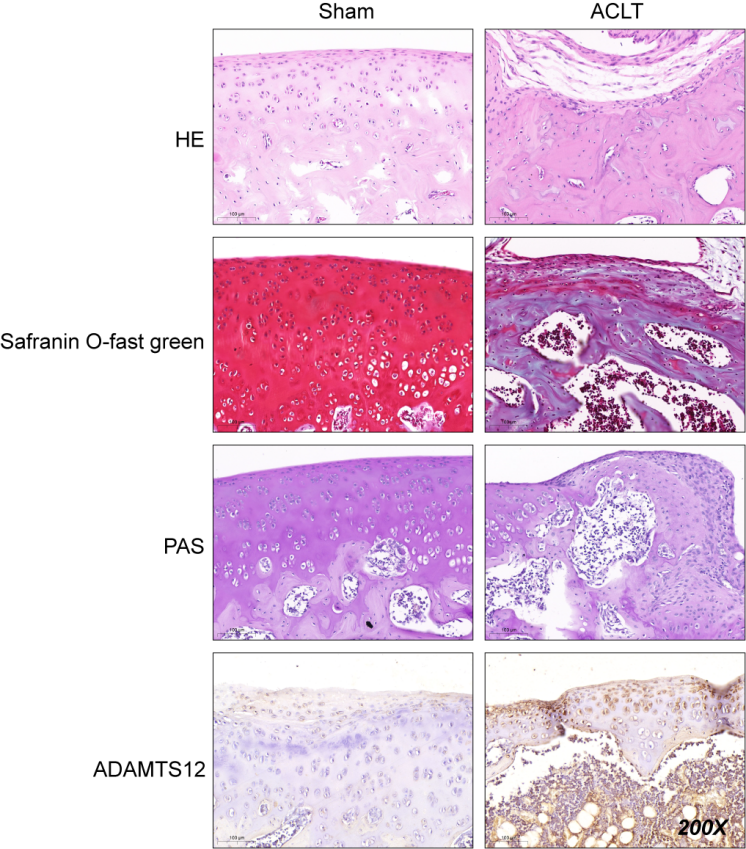
**

**Figure S2. Establishment of OA animal model with improved ADAMTS12 expression.** OA rats were induced by ACL-T method. HE, PAS, safranin O-fast green and IHC (ADAMTS12) of cartilage tissues in each group. n = 5.


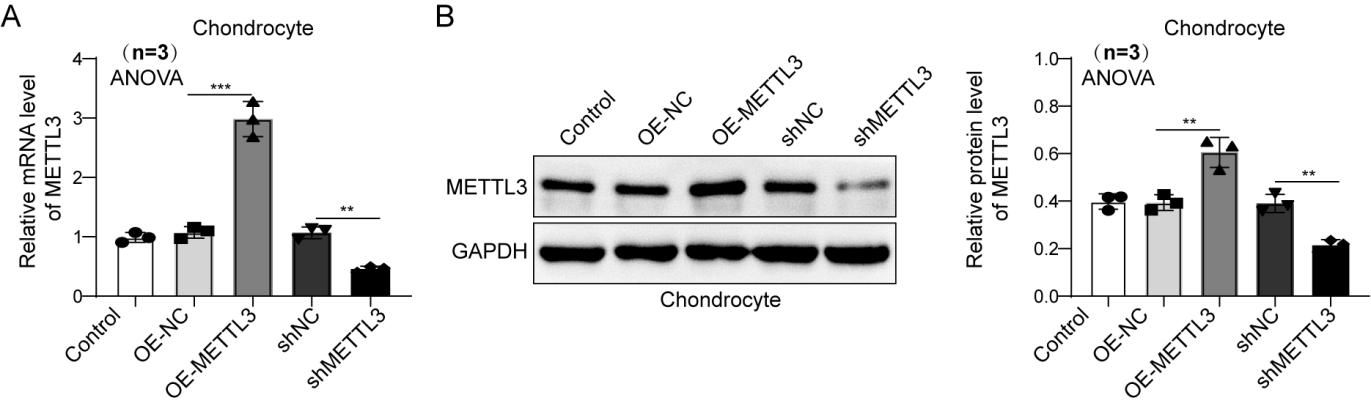


**Figure S3. Over-expression and knockdown efficiency of METTL3 in rat chondrocytes.** (A) Relative mRNA expression level of METTL3 detected by RT-qPCR. n = 3 (B) Relative protein expression level of METTL3 detected by western blot. n = 3. **, *P* < 0.01; ***, *P* < 0.001.


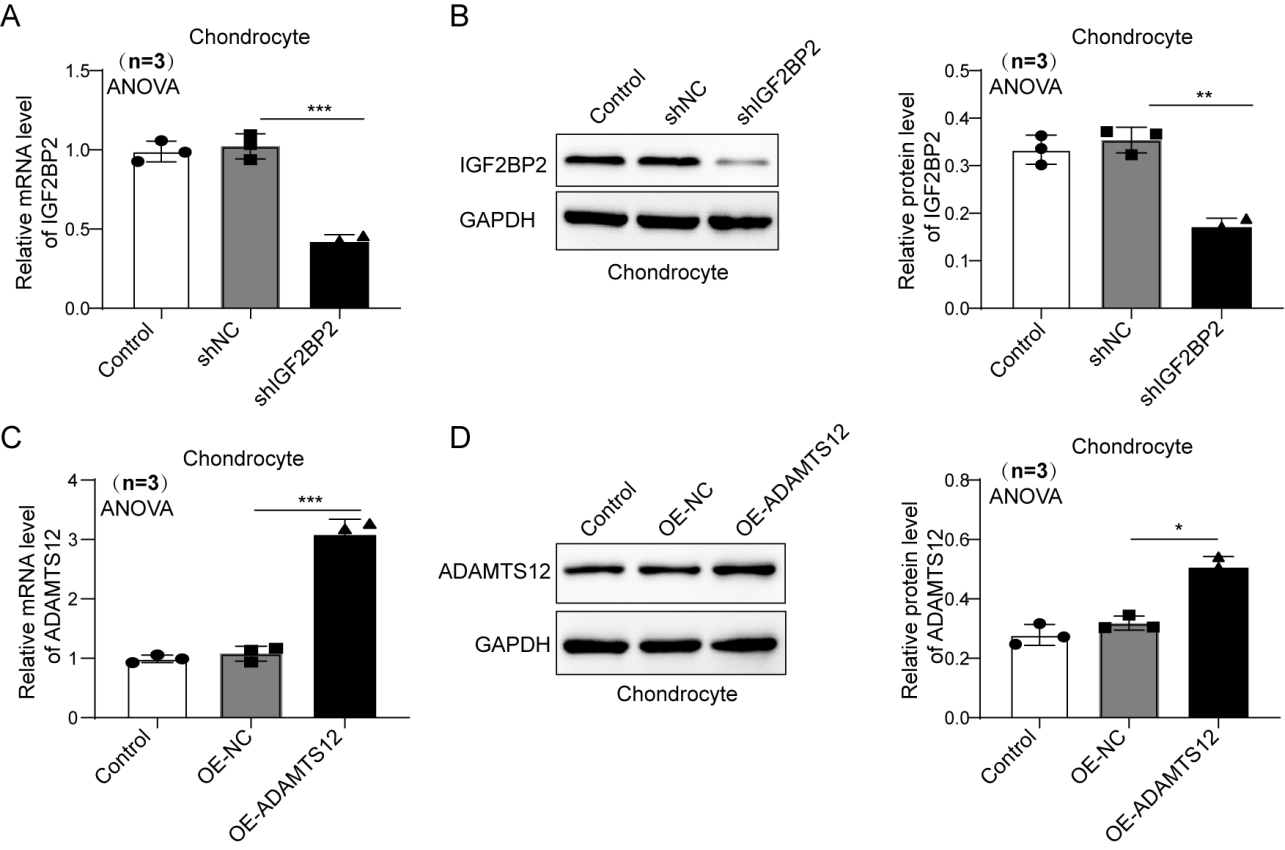


**Figure S4.** Knockdown efficiency of IGF2BP2 in rat chondrocytes through RT-qPCR (A) and western blotting (B). n = 3. Over-expression efficiency of ADAMTS12 in chondrocytes through RT-qPCR (C) and western blotting (D). n = 3. **P* < 0.05; **, *P* < 0.01; ***, *P* < 0.001.
